# Chlorination of Clothianidin During Disinfection: Kinetics, Pathways, and Toxicity

**DOI:** 10.3390/toxics14060453

**Published:** 2026-05-22

**Authors:** Fang Wei, Lei Wu, Fei Meng, Sanyan Du, Xinyuan Wu, Jun Hu

**Affiliations:** 1 Department of Hydraulic Engineering, Zhejiang Tongji Vocational College of Science and Technology, Hangzhou 310000, China; 2 Longyou Inspection & Testing Research Institute, Quzhou 324000, China; 3 Zhejiang Yilong Environmental Protection Technology Co., Ltd., Hangzhou 310000, China; 4 Yangtze River Delta Institute of Health Agriculture (Zhejiang) Co., Ltd., Jiaxing 314000, China; 5 College of Environment, Zhejiang University of Technology, Hangzhou 310000, China

**Keywords:** clothianidin, chlorination, kinetics, pathways, toxicity

## Abstract

Neonicotinoid pesticides are a typical category of emerging hazardous micropollutants, and chlorine (Cl_2_) is a widely used disinfectant that readily induces the chlorination of organic micropollutants. This study systematically investigated the chlorination kinetics and transformation pathways of a representative neonicotinoid pesticide (clothianidin, CLO) and evaluated the cytotoxicity variation via Chinese Hamster Ovary (CHO) cell assays. CLO chlorination followed second-order kinetics, with a first-order dependence on both CLO and Cl_2_ concentrations, and the apparent rate constant (*k*_app_) value was measured to be 1.758 × 10^−4^ μM^−1^ h^−1^, at a pH of 7.0. The CLO chlorination initially accelerated and then retarded with the increase in pH. The same tendency was involved in the yield of disinfection byproducts (i.e., trihalomethanes and haloacetic acids). Dissolved organic matter was also a crucial factor inhibiting the chlorination of CLO. The reaction of CLO^+^ with HOCl was more prevalent than between CLO^+^ with ClO^−^, wherein HOCl likely exerts electrophilic attack either after 2-nitroguanidine hydrolysis or directly at the nitrogen sites of secondary amines. Cell exposure results revealed that the chronic cytotoxicity of CLO decreased significantly after chlorination. This study helps to the mechanistic understanding of neonicotinoid transformation during water disinfection, and provides a valuable reference for the control of neonicotinoid pesticides in drinking water.

## 1. Introduction

Neonicotinoids are the fourth generation of insecticides following organophosphates, carbamates and pyrethroids and have become the linchpin in the integrated management of agricultural pests worldwide [1,2]. Clothianidin (CLO) is a typical second-generation neonicotinoid that has risen to become one of the most prevalently used insecticides [3]. The overuse of CLO has led to its discharge into aquatic environments, thereby posing potential negative impacts on ecosystems and human health, particularly due to its neurobehavioral effects [4,5,6,7]. CLO is one of the neonicotinoids with the highest detection frequency in drinking water sources due to its relatively high hydrophilicity [3,7,8]. It was reported that CLO ranked as the second-most prevalent neonicotinoid in the tributaries of the Great Lakes, the largest freshwater ecosystem around the world [9]. In an extensive groundwater survey in China, CLO exists at a maximum concentration of 137 ng L^−1^ [10]. Moreover, CLO cannot be completely removed and persists in finished water and tap water worldwide, even after treatment at waterworks and transportation by pipelines [8,11,12,13,14].

As is well known, the disinfection process plays a crucial and indispensable role in controlling pathogenic microorganisms in drinking water. To date, chlorine is still regarded as the most prevalent chemical oxidant for drinking water disinfection globally, owing to its excellent bactericidal ability and low cost [15]. Typically, the disinfection process involves the chlorine application (several mg L^−1^) at one or two specific points, including pre-treatment (to conduct primary disinfection at the beginning of the water treatment process) and/or post-treatment (to maintain a disinfectant residual in water pipes) [16]. Nevertheless, chlorine usually reacts with the micropollutants in water, inducing the formation of highly toxic disinfection byproducts (DBPs) [17,18,19,20,21].

Currently, there are few in-depth reports on the neonicotinoid chlorination, except for imidacloprid (IMI). As reported by Klarich et al. [8], CLO was more susceptible to reaction with chlorine than IMI, with half-lives of ca. 2.5 and 70 days, respectively, at a chlorine residual of 5.0 mg Cl_2_ L^−1^. Through the identification of chlorination products (CPs), the reaction pathways of IMI were found to involve the direct chlorination of the parent compound and the indirect chlorination of its metabolites (i.e., imidacloprid-urea and desnitro-imidacloprid) [22]. Yet, the reaction kinetics and changes in toxicity had not been systematically elucidated, which was a concern in the field of drinking water health.

The aim of this study was to comprehensively investigate the chlorination process of CLO and evaluate the changes in toxicity. The issues put emphasis on: (1) exploring the chlorination kinetics under different pH conditions; (2) investigating the formation of typical DBPs during chlorination; (3) proposing possible chlorination pathways of CLO; (4) illustrating the changes in chronic cytotoxicity after chlorination using Chinese Hamster Ovary (CHO) cells. This study enhances the understanding of neonicotinoid conversion during the disinfection process and offers a valuable reference for neonicotinoid control in drinking water.

## 2. Materials and Methods

### 2.1. Chemical Reagents

All chemicals used in this study were of ACS grade or higher. Free chlorine (Cl_2_) was spiked as a standardized sodium hypochlorite solution (NaOCl, >10%) (Sinopharm Chemical Reagent, Shanghai, China). CLO and Na_2_S_2_O_3_ were purchased commercially from TMstandard (Jiangsu, China) and Sigma-Aldrich (Shanghai, China), respectively. Sodium carbonate (Na_2_CO_3_) and sodium nitrate (NaNO_3_) were obtained from Sinopharm Chemical Reagent Company (Shanghai, China), and humic acid was purchased from Sigma-Aldrich (Shanghai, China). The humic acid stock solution was preliminarily prepared by dissolving the solids in deionized water and then filtering through 0.45-μm membrane filters. Three real waters were sampled from the waterworks in Huzhou, Jinhua and Nanchang cities (Table S1).

### 2.2. Experimental Procedure

All experimental procedures were performed in a dark environment at a controlled room temperature of 25 °C, with continuous magnetic stirring at a speed of 400 rpm. Prior to use, the reaction bottles were soaked in NaClO solution for 2 h and then thoroughly rinsed three times with Milli-Q water to ensure no chlorine demand. The pH of the reaction solutions was adjusted using phosphate buffer (10 mM). Notably, a negligible change in pH (±0.1) was observed after the reaction. To cover a wide range of real water conditions as comprehensively as possible, the initial pH of the solution was deliberately set between 6.0 and 9.5. NaClO solution was added to the buffer solution containing CLO. Given that the Cl_2_ concentration was significantly higher than that of CLO during the disinfection process, the initial concentration ratio of Cl_2_ to CLO (denoted *R*_Cl/CLO_) was precisely controlled between 100:1 and 800:1. This was done to ensure an excess of Cl_2_ in the reaction system. Meanwhile, CLO concentration was increased to 0.1 mg L^−1^ to facilitate accurate detection and analysis. As the reaction proceeded, water samples were collected systematically at regular time intervals. After sampling, an excess of Na_2_S_2_O_3_ was quickly added to scavenge residual chlorine effectively. Subsequently, the samples were filtered through 0.22-μm membranes before quantification. The chronic cytotoxicity test was conducted using CHO cells (Text S1). CHO cells were purchased from Zhejiang Ruyao Biotech Co., Ltd. (Ningbo, China) and are archived in Cellosaurus database (accession number: CVCL_7183). And each experiment was repeated at least twice to ensure the reliability and reproducibility of the results. Additionally, relative percent differences were calculated to evaluate the consistency and variability among experimental replicates quantitatively.

### 2.3. Analytical Methods

CLO was analyzed using an ultra-high performance liquid chromatograph coupled with a tandem mass spectrometer (UPLC-MS/MS, Waters Xevo TQ-s, Milford, MA, USA) and a YMC-Pack ODS-AQ column (2.1 mm × 100 mm, 3.0 μm) (YMC, Kyoto, Japan). Trihalomethanes (THMs) and haloacetic acids (HAAs) were quantified using a gas chromatograph integrated with an electron capture detector (GC/ECD, Agilent 6890N) and a DB-5 ms capillary column (30 m × 0.25 mm, 0.25 μm) (Agilent, Santa Clara, CA, USA). The chlorination products were identified by an ultra-performance liquid chromatography integrated with a quadrupole time-of-flight mass spectrometer (UPLC-QTOF-MS/MS, ACQUITY UPLC I-Class, Waters SYNAPT XS HDMS) and a Waters ACQUITY BEH C18 column (2.1 mm × 1000 mm, 1.7 μm) (Waters, Milford, MA, USA). Nitrate (NO_3_^−^) and nitrite (NO_2_^−^) were measured by a Dionex Aquion RFIC ion chromatograph with an IonPac AS19 column (250 mm × 4.0 mm, 5.0 μm), and ammonium (NH_4_^+^) was analyzed by a Dionex ICS-3000 ion chromatograph with an IonPac CS12A column (250 mm × 4.0 mm, 5.0 μm) (Thermo Fisher Scientific, Waltham, MA, USA). The residual chlorine was determined spectrophotometrically using the *N*,*N*-diethyl-*p*-phenylenediamine method [23]. The relative instrumental parameters and operational procedures are provided in Text S2.

## 3. Results and Discussion

### 3.1. Time-Dependent Chlorination of CLO

**Figure 1:**
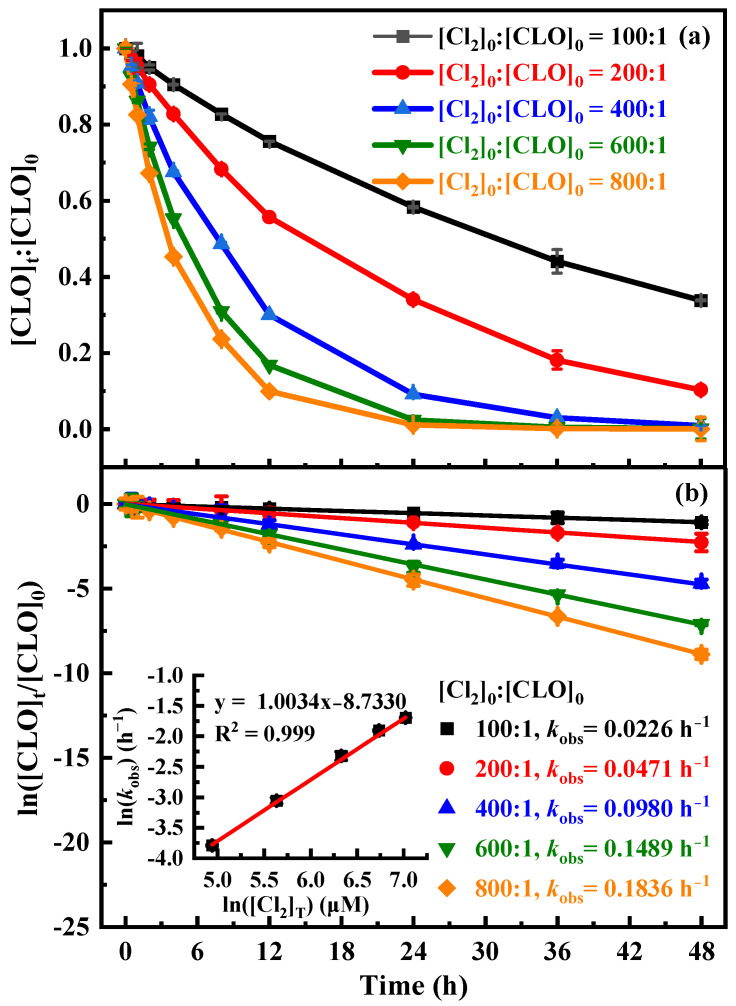
Kinetics of clothianidin (CLO) chlorination: (**a**) degradation curves; (**b**) kinetic fitting. Experimental conditions: [CLO]_0_ = 0.1 mg L^−1^, pH = 7.0.

In this section, the chlorination of CLO was conducted under the condition of pH 7.0. The hydrolysis of CLO was negligible over a span of 36 h (Figure S1). Obviously, CLO chlorination accelerated gradually as the *R*_Cl/CLO_ value was elevated from 100:1 to 800:1 (Figure 1a). Additionally, the consumption of Cl_2_ (Δ[Cl_2_]) was measured to be −1.0 μM, which accounted for a mere 0.7% of the initial dosage (Figure S2). This finding clearly demonstrates that Cl_2_ was present in a substantial excess relative to CLO, even when the *R*_Cl/CLO_ value was 100:1. Therefore, the Cl_2_ concentration can be reasonably assumed to remain constant throughout the reaction.

As illustrated in Figure 1b, as the *R*_Cl/CLO_ value was increased from 100:1 to 800:1, the observed rate constant (*k*_obs_) of CLO increased from 0.0226 to 0.1836 h^−1^ (Equation (1)). Moreover, the ln(*k*_obs_) values exhibited a linear correlation with the ln([Cl_2_]_T_) values, and the slope (i.e., *m*) was measured to be 1.0034, through the linear regression analysis (Text S3). This result unambiguously demonstrates that the chlorination of CLO followed second-order kinetics, with a first-order dependence on both CLO and Cl_2_ concentrations (Equation (2)). Afterwards, the apparent rate constant (*k*_app_) was determined to be 1.758 × 10^−4^ μM^−1^ h^−1^ (Equation (3)). It is worth noting that the *k*_app_ value is inherently independent of absolute Cl_2_ and CLO concentrations under excess chlorine conditions. Thus, the simultaneous increase in the concentrations of Cl_2_ and CLO did not interfere with the kinetic analysis of CLO chlorination.

(1) $$-\frac{d[\mathrm{CLO}]}{\mathrm{dt}}=k_{\mathrm{obs}}{\left[\mathrm{CLO}\right]}_{T}=k_{\mathrm{app}}{\left[{\mathrm{Cl}}_{2}\right]}_{T}^{m}{\left[\mathrm{CLO}\right]}_{T}$$

(2) $$-\frac{d[\mathrm{CLO}]}{\mathrm{dt}}=k_{\mathrm{obs}}{\left[\mathrm{CLO}\right]}_{T}=k_{\mathrm{app}}{\left[{\mathrm{Cl}}_{2}\right]}_{T}{\left[\mathrm{CLO}\right]}_{T}$$

(3) $$k_{\mathrm{app}}=\frac{k_{\mathrm{obs}}}{{\left[{\mathrm{Cl}}_{2}\right]}_{T}}$$

### 3.2. pH-Dependent Reaction Kinetics

**Figure 2:**
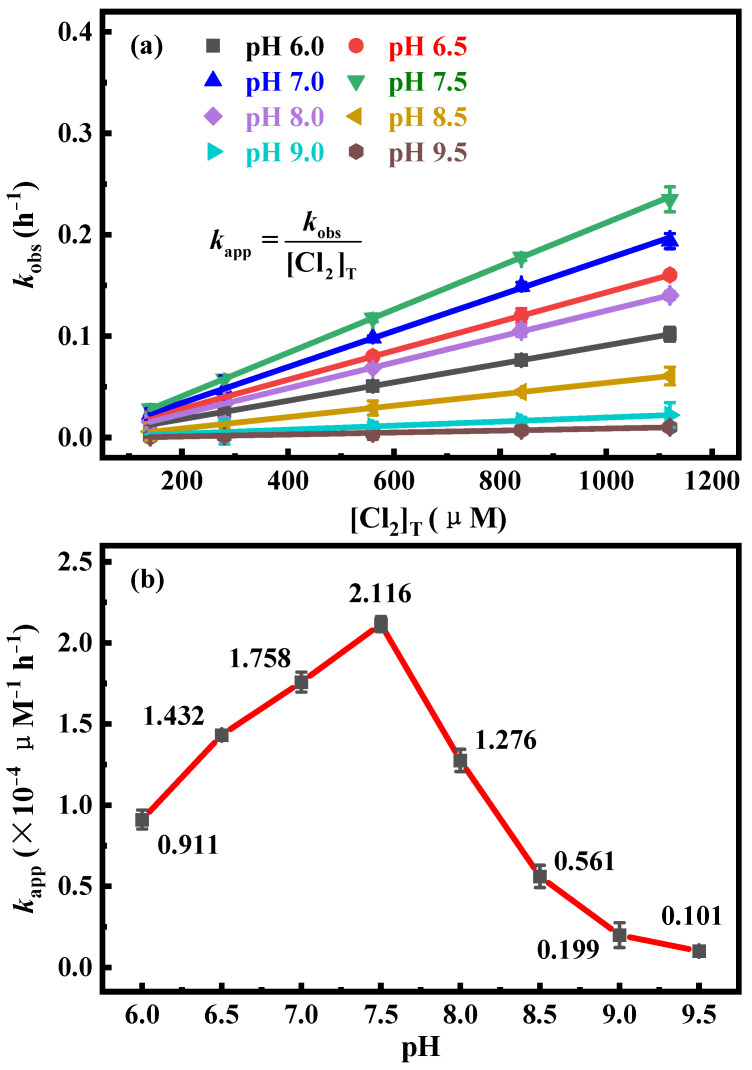
pH-dependent reaction kinetics of CLO chlorination: (**a**) *k*_obs_ values; (**b**) *k*_app_ values. Experimental conditions: [CLO]_0_ = 0.1 mg L^−1^.

The chlorination kinetics of CLO were investigated within the pH range of 6.0 to 9.5. The CLO chlorination initially accelerated and then retarded with increasing pH (Figure S3 and Figure 2a). Specifically, the *k*_app_ value increased from 0.911 × 10^−4^ to 2.116 × 10^−4^ μM^−1^ h^−1^ as the pH was increased from 6.0 to 7.5, while significantly decreased to 0.101 × 10^−4^ μM^−1^ h^−1^ when the pH was further raised to 9.0 (Figure 2b).

Within the given pH range, CLO (p*K*_a_ = 11.0) underwent complete protonation and mainly existed as a cationic species (CLO^+^), based on the theoretical calculation using the Henderson–Hasselbalch equation (Equation (4)) [24]. It is a well-established fact that the aqueous chlorine was primarily present as hypochlorous acid (HOCl) and hypochlorite ion (ClO^−^) under the given pH condition [16]. Consequently, it is reasonably hypothesized that the CLO chlorination was induced by the reactions of CLO^+^ with HOCl and OCl^−^. In view of the extremely low *k*_app_ value at pH 9.6, it can be inferred that the reaction between CLO^+^ and OCl^−^ was relatively inert, and the reaction of CLO^+^ with HOCl was more prevalent. Text S4 describes the second-order reaction kinetics of CLO chlorination, based on the preliminary idealized assumption that only these two reaction pathways existed and proceeded independently of each other. According to Equation (5), the *k*_app_ value should continuously decrease with increasing pH, given that the reaction between CLO^+^ and HOCl is thermodynamically and kinetically favored over the reaction between CLO^+^ and OCl^−^. However, the experimental results show that the *k*_app_ value exhibited an initial increase followed by a significant decrease as pH rose, which clearly deviates from the idealized model prediction. Regrettably, it remains challenging to definitively clarify the fundamental mechanism underlying this inconsistency. A plausible hypothesis to rationalize this discrepancy is the potential synergistic interaction between the two reaction pathways. Specifically, acidic substances generated from the reaction of CLO^+^ with HOCl may neutralize the alkaline species produced from the reaction of CLO^+^ with OCl^−^, thereby establishing a favorable local acid–base environment for HOCl-mediated oxidation. Such a potential synergistic tendency seems to be most prominent at pH 7.6. At excessively high pH, the chlorination reaction was dominated by low-reactivity OCl^−^, which inevitably lowered the overall reaction rate. In comparison, an extremely low OCl^−^ fraction at low pH cannot provide sufficient complementary contribution to sustain the synergistic interaction. Apart from the possible synergistic effect, this inconsistency can also be attributed to several other unconsidered factors, including the pH-dependent speciation transformation and structural conformation variation in the CLO molecule, as well as the possible participation of intermediate reactive species that have not been incorporated into the simplified kinetic model. The actual chlorination behavior of CLO under varying pH conditions was governed by complex coupled reactions and multiple interactive factors, which cannot be fully reproduced by the idealized and oversimplified assumptions adopted in the kinetic model.

(4) $$\mathrm{pH}=pK_{a}+\mathrm{lg}\frac{\left[\mathrm{CLO}\right]}{\left[{\mathrm{CLO}}^{+}\right]}$$

(5) $$k_{\mathrm{app}}=\frac{k_{\mathrm{HOCl},{\mathrm{CLO}}^{+}}[H^{+}]+k_{{\mathrm{OCl}}^{-},{\mathrm{CLO}}^{+}}K_{a}^{\mathrm{HOCl}}}{K_{a}^{\mathrm{HOCl}}+[H^{+}]}$$

### 3.3. Impact Factors

**Figure 3:**
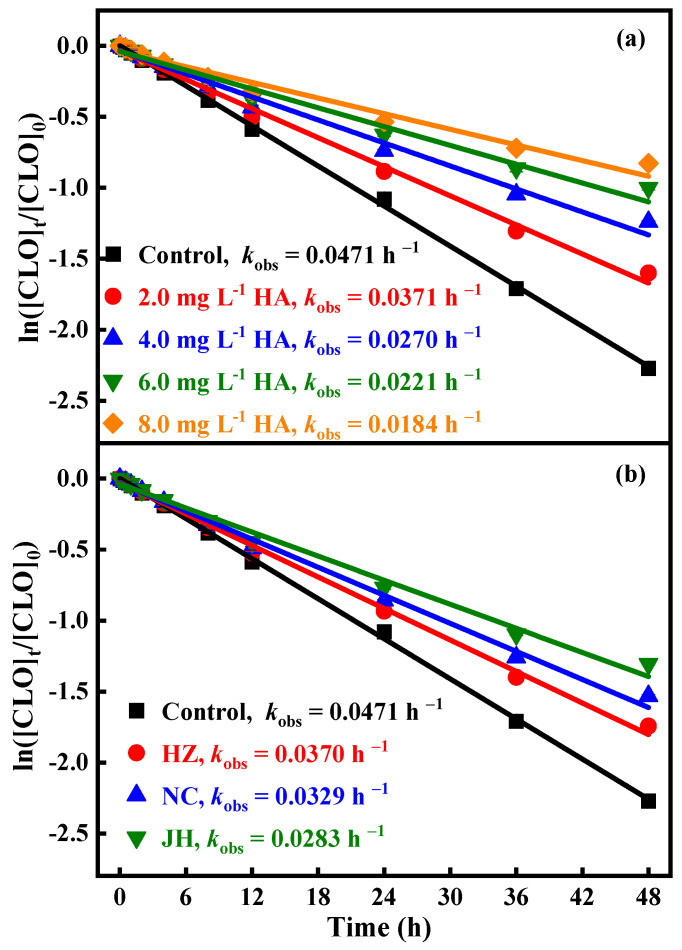
Effect of dissolved organic matter (**a**) and real waters (**b**) on the chlorination of CLO. Experimental conditions: [Cl_2_]_0_ = 20 mg L^−1^, [CLO]_0_ = 0.1 mg L^−1^, pH = 7.0. HA: humic acid; HZ, NC, and JH: the water samples collected from the waterworks in Huzhou, Nanchang and Jinhua cities (China).

In this study, the impact of typical inorganic anions and dissolved organic matter on the chlorination of CLO was investigated and verified in three real waters. As shown in Figure S4a,b, two inorganic anions, CO_3_^2−^ and NO_3_^−^, had no visible impact on the chlorination of CLO. However, it does not mean that all inorganic anions will not affect CLO chlorination, because those inorganic anions with reducibility (e.g., sulfite SO_3_^2−^; cyanide, CN^−^; and iodide, I^−^) may consume aqueous chlorine, thus retarding the chlorination of CLO [16]. By the same token, the presence of humic acid (a surrogate of dissolved organic matter) inhibited the CLO chlorination as expected. The *k*_obs_ value decreased from 0.0471 to 0.0184 h^−1^ as the humic acid content increased to 8.0 mg L^−1^, with the *R*_Cl/CLO_ value of 200:1 (Figure 3a and Figure S4c). The three selected real water matrices exhibited similar pH and SUVA_254_ values (Table S1). Since SUVA_254_ is a representative indicator reflecting DOM aromaticity and humic-like composition, the negligible difference in SUVA_254_ among the three water samples indicates their analogous DOM structural characteristics. Such narrow variation in SUVA_254_ precludes a reliable quantitative correlation between DOM compositional properties and kinetic parameters. Nevertheless, the three real waters displayed obvious differences in overall DOM concentration, enabling a valid quantitative analysis between DOM content and chlorination kinetics. Consistent with the humic acid results, apparent inhibition of CLO chlorination was also observed in all three real water samples. The inhibitory effect gradually intensified with increasing DOM concentration, accompanied by a continuous decline in *k*_obs_ value (Figure 3b and Figure S4d). It confirms that DOM acted as an important competitor for chlorine, and thus suppressed the chlorination of CLO in natural water matrices.

### 3.4. Nitrogen Release and DBPs Formation

The nitrogen salts, such as NH_4_^+^, NO_2_^−^ and NO_3_^−^, were monitored simultaneously to explore the nitrogen release during the chlorination of CLO. It was clearly observed that only NO_3_^−^ was generated, with neither NH_4_^+^ nor NO_2_^−^ formed throughout the CLO chlorination process (Figure S5). Unsurprisingly, pH had a pronounced influence on the formation of NO_3_^−^, indicating a congruent trend with its effect on the CLO chlorination. The NO_3_^−^ formation increased first and then decreased with increasing pH, reaching a maximum yield of 16.9% at pH 7.5.

**Figure 4:**
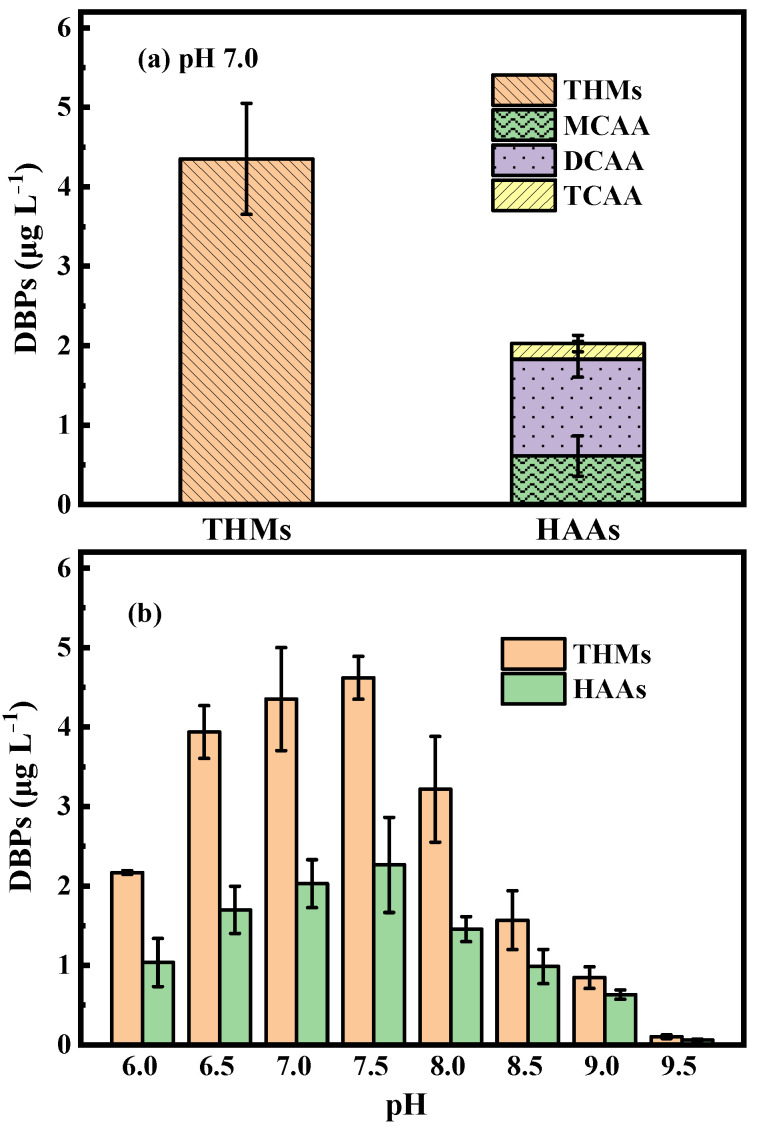
Formation of disinfection byproducts (DBPs): (**a**) formation of THMs and HAAs at pH 7.0; (**b**) effect of pH on the formation of THMs and HAAs. Experimental conditions: [Cl_2_]_0_ = 10 mg L^−1^, [CLO]_0_ = 0.1 mg L^−1^. THMs, trihalomethanes; HAAs, haloacetic acids; MCAA, monochloroacetic acid; DCAA, dichloroacetic acid; TCAA, trichloroacetic acid.

Considering the strict global regulations on DBPs, the formation of two common DBPs (i.e., THMs and HAAs) was investigated in this study. THMs and HAAs only include chlorinated DBPs, as the reaction system excluded all halogen species except chlorine. Accordingly, THMs were defined as trichloromethane (TCM), while HAAs included mono-, di- and tri-chloroacetic acid (MCAA, DCAA and TCAA, respectively) in this study. During CLO chlorination, the yields of THMs and HAAs were quantified and found to be quite low, specifically less than 5.0 μg L^−1^ (Figure 4a). The formation potential of THMs was relatively higher than that of HAAs. After 48 h of chlorination, the yields of THMs and HAAs were measured to be 4.4 and 2.0 μg L^−1^, respectively. As illustrated in Figure 4b, as the pH increased, the DBP yields first showed an upward trend and then a downward one, with the maximum achieved at a pH of 7.5. In addition, the HAAs species also changed with pH variation. The average number (*n*) of chlorine atom was used to denote the substitution degree of HAAs (Equation (6)).

(6) $$n=\frac{\left[\mathrm{MCAA}]+2[\mathrm{DCAA}]+3[\mathrm{TCAA}\right]}{\left[\mathrm{MCAA}]+[\mathrm{DCAA}]+[\mathrm{TCAA}\right]}$$

As the pH was elevated from 6.0 to 8.5, the *n* value first increased from 1.75 to 1.83, and then decreased to 1.72 (Figure S6). The results regarding the yields and species of DBPs were consistent with the effect exerted by pH on CLO chlorination. Liang and Singer [25] conducted an in-depth analysis of the influence of pH on the formation of THMs and HAAs. A definite conclusion was drawn that the base-catalyzed hydrolysis prevailed under alkaline conditions and thereby produced more THMs, while HAAs were more likely to be generated in acidic environments due to the stronger oxidation capacity of HOCl. The discrepant results with our study reveal that the initial chlorination process affected by pH may be the determining step in the formation of DBPs from CLO.

### 3.5. Reaction Pathways

**Figure 5:**
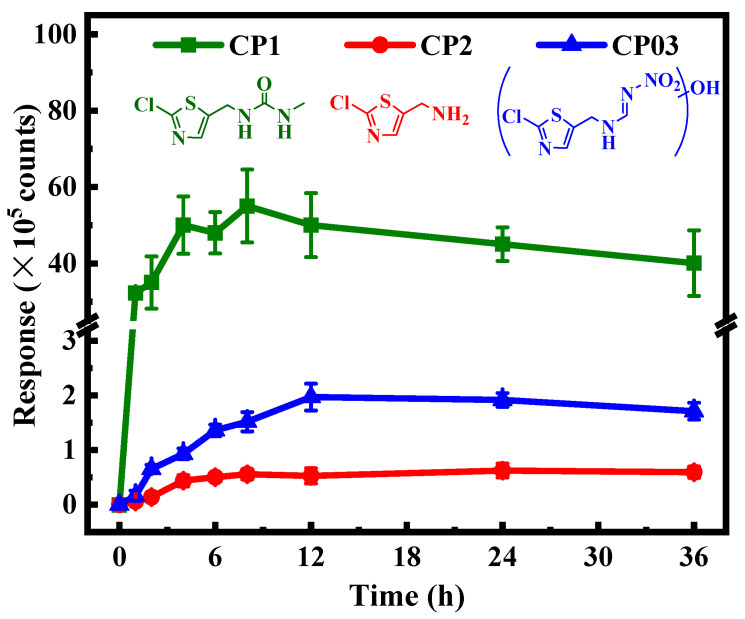
Temporal progression of identified chlorination products of CLO. Experimental conditions: [Cl_2_]_0_ = 10 mg L^−1^, [CLO]_0_ = 0.1 mg L^−1^, pH = 7.0.

The reaction products of CLO were tentatively identified via UPLC-QTOF-MS/MS. Briefly, experimental fragment ion spectra were interpreted by reference to theoretically predicted ionization and fragmentation characteristics. The mass error was controlled within 3 mDa for both precursor and product ions. The experimental isotopic abundance patterns were matched against theoretical distributions to ensure the reliability of product identification (Text S2). Consequently, three reaction products were detected for the CLO chlorination (Table S2 and Figures S7–S9), and their time-course production is illustrated in Figure 5. Surprisingly, no chlorinated products were detected in this study, which may be ascribed to ultra-low generation levels of potential chlorinated intermediates, mutual mass spectral interference from coexisting components and rapid degradation of unstable intermediates. Moreover, authentic chemical standards for potential chlorinated intermediates are unavailable, which makes it difficult to determine their exact detection thresholds and confirm their presence.

**Figure 6:**
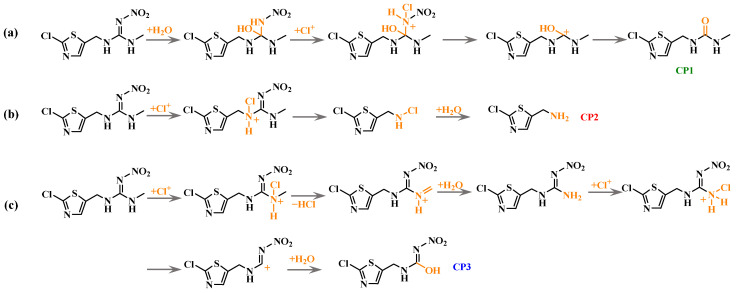
Proposed chlorination pathways of CLO: (**a**) CP1 (green); (**b**) CP2 (red); (**c**) CP3 (blue). Experimental conditions: [Cl_2_]_0_ = 10 mg L^−1^, [CLO]_0_ = 0.1 mg L^−1^, pH = 7.0.

The chemical structure of HOCl was characterized by the polarization of the Cl-O bond (Cl*^δ^*^+^-OH*^δ^*^−^), which rendered the electrophilic chlorine moiety (Cl^+^) to preferentially attack electron-rich groups, such as unsaturated bonds, nitrogen-containing functionalities, and sulfur-containing groups [16]. Combined with the mass spectral identification results, the reaction of CLO with HOCl is proposed to proceed via the following pathways: One plausible pathway initiated with the hydrolysis of the 2-nitroguanidine moiety to yield an *α*-hydroxylamine group (-C(OH)-NH-). Subsequent electrophilic attack by Cl^+^ produced a positively charged N-chloro-α-hydroxylamine intermediate (-C(OH)-NCl-). This unstable intermediate underwent intramolecular charge rearrangement, in which the positive charge migrated from the nitrogen atom to the central carbon atom. The C-N bond then cleaved to form a positively charged hydroxyl-substituted carbon intermediate (-C-OH). Thereafter, dehydration and structural rearrangement of the hydroxyl group (-OH) occurred to form a carbonyl group (-C=O), which corresponds to CP1 and possesses a typical urea skeleton structure (Figure 6a). A second pathway involved the electrophilic substitution of secondary amine sites (-NH-) to produce positively charged *N*-chloramine species (-NCl-). Due to the presence of two inequivalent secondary nitrogen atoms in the 2-nitroguanidine structure, two reaction branches were available for this electrophilic substitution. In the first branch, Cl^+^ preferentially attacked the secondary nitrogen adjacent to the thiazole ring. The resulting *N*-chloramine underwent heterolytic cleavage of the C-N bond, where charge redistribution occurred synchronously with bond breaking without prior intramolecular rearrangement. The newly formed *N*-chloramine was further hydrolyzed to produce a primary amine (-NH_2_), assigned as CP2 (Figure 6b). In the second branch, the electrophilic attack took place at the secondary nitrogen on the opposite side of the 2-nitroguanidine group. The corresponding *N*-chloramine sequentially underwent dehydrochlorination and *N*-dealkylation to produce a primary amine [26]. This primary amine was further subjected to another electrophilic attack by Cl^+^ to form a new *N*-chloramine intermediate. The intermediate subsequently underwent heterolytic C-N bond cleavage to yield a resonance-stabilized carbocation, which was then hydrolyzed to form an enol-like structure (-C(OH)=N-) corresponding to CP3 (Figure 6c). Notably, mass spectrometry alone cannot unambiguously determine the exact substitution position of the hydroxyl group. Nevertheless, based on the pathway deduction, CP3 is most plausibly assigned to this enol-like structural configuration. In fact, the sulfur atom on the thiazole ring is thermodynamically susceptible to oxidation by HOCl, potentially forming sulfoxide and sulfone derivatives, as reported in our previous work [27]. However, no sulfur-oxidized products were experimentally detected in the present study; thus, the related oxidation pathway was not proposed herein. In addition, CP1 was also identified in the UV/Cl_2_ treatment system reported by Lee et al. [28], whereas CP2 and CP3 were not observed. Such discrepancies can be reasonably attributed to the fact that radical reactions dominate the UV/Cl_2_ process, while the HOCl oxidation system in this study is governed mainly by electrophilic substitution reactions.

### 3.6. Mammalian Cell Toxicology

**Figure 7:**
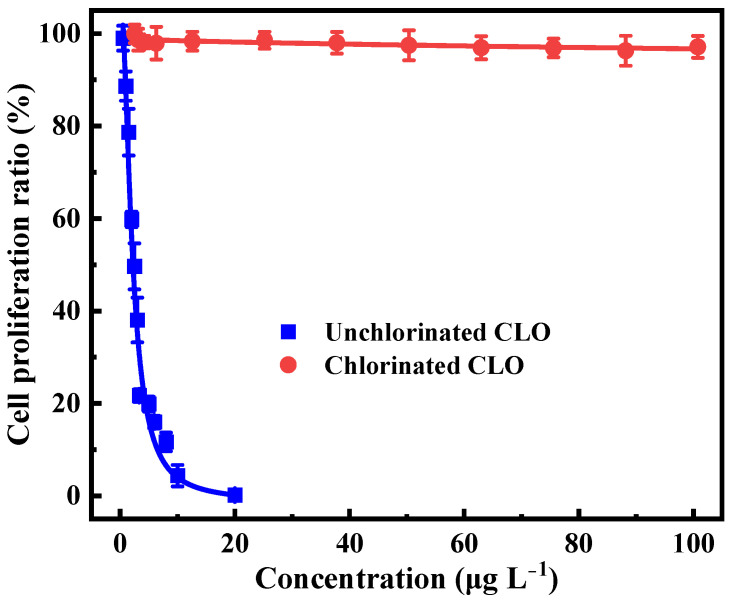
Concentration–response curves of the chlorinated samples and the unchlorinated negative control. Experimental conditions: [Cl_2_]_0_ = 10 g L^−1^, [CLO]_0_ = 0.1 g L^−1^, pH = 7.0.

To enable the accurate quantification of chlorination products, the concentrations of CLO and Cl_2_ were increased by a thousand-fold (to 0.1 and 10 g L^–1^, respectively). As a result, the yield of chlorination products was determined to be 0.13 g L^–1^ (Text S1). Based on the appropriate concentrations determined in the preliminary experiment, it was decided to expose CHO cells to the unchlorinated and chlorinated CLO solutions after dilution. The chlorinated CLO solution was diluted 1250–50,000 fold to yield a product concentration range of 2.5–100 μg L^−1^. The unchlorinated CLO for negative control was prepared at a concentration range of 0.5–20 μg L^−1^. As illustrated in Figure 7, the chlorination process resulted in a substantial reduction in the chronic cytotoxicity of CLO against CHO cells. Such a qualitative trend is independent of the initial CLO concentration. Moreover, the concentration–response curves were plotted based on the cell proliferation ratios of CHO cells and the concentrations of CLO and its chlorination products, which can also provide a valuable reference for evaluating the product toxicity at environmentally relevant levels. Through the regression analysis, the median lethal concentration (LC_50_) value of unchlorinated CLO was calculated to be 2.1 μg L^−1^. In contrast, the LC_50_ value of chlorinated CLO could not be obtained due to the insufficient data caused by its considerably lower toxicity. Given the absence of previous reports on the toxicity changes during CLO chlorination, references can only be drawn from other chemical processes. Todey et al. [29] reported that the photolytic products of CLO exhibited no residual toxicity to mosquito (*Culex pipiens*) larvae. During the photo-catalytic process, humic acid, acting as a photosensitizer, induced the formation of low-toxicity CLO products, leading to a 50% reduction in the bioluminescence inhibition of *Vibrio fischeri* [30]. In a heterogeneous electro-Fenton system coupled with three-dimensional electrodes fabricated from a biochar-supported zero-valent iron nanoparticle hybrid material, CLO degradation showed a pronounced tendency to reduce toxicity to *Daphnia* and green algae [3]. However, in such a system, the dechlorination pathway initiated by zero-valent iron was of utmost importance for reducing the toxicity of CLO, which is quite different from the situation in this study as the non-detection of dechlorinated products.

Overall, the chlorine-based disinfection seems to contribute to the detoxification of CLO. However, as mentioned above (Section 3.3), chlorine can react with numerous inorganic and organic matters present in water, which may also exert an impact on the species composition and toxicity characteristics of the chlorinated products of CLO.

## 4. Conclusions

This study investigated the pH-dependent chlorination kinetics of CLO, the reaction pathways of CLO chlorination, and the corresponding changes in chronic cytotoxicity using CHO cells. The conclusions are drawn as follows, according to the experimental results:

- The CLO chlorination followed second-order kinetics, with a first-order dependence on both CLO and Cl_2_ concentrations. The *k*_app_ value was 1.758 × 10^−4^ μM^−1^ h^−1^, at pH 7.0.
- The chlorination of CLO accelerated initially and then retarded with increasing pH. The same tendency was involved in the formation of DBPs (THMs and HAAs).
- The reaction of HOCl with CLO^+^ prevailed, wherein HOCl likely exerts electrophilic attack either after 2-nitroguanidine hydrolysis or directly at the nitrogen sites of secondary amines.
- The LC_50_ of unchlorinated CLO to CHO cells was measured to be 2.1 μg L^−1^, and its chronic cytotoxicity decreased significantly after chlorination.

## Data Availability

The datasets presented in this article are not readily available due to technical limitations. Requests to access the datasets should be directed to the corresponding author.

## Supplementary Materials

The following supporting information can be downloaded at: https://www.mdpi.com/article/10.3390/toxics14060453/s1, Text S1: Chronic cytotoxicity assay; Text S2: Instrumental conditions for chemical analysis [31,32]; Text S3: Determination of reaction order (*m*); Text S4: pH-dependent reaction kinetics of CLO chlorination; Table S1: Major characteristics of real waters post 0.22 μm membrane filtration; Table S2: Identified products of clothianidin (CLO); Figure S1: Hydrolysis of clothianidin (CLO). Experimental conditions: [NPs]_0_ = 0.1 mg L^−1^, pH = 7.0; Figure S2: Chlorine consumption during CLO chlorination. Experimental conditions: [Cl_2_]_0_ = 10 mg L^−1^ (0.14 mM), [CLO]_0_ = 0.1 mg L^−1^, pH = 7.0; Figure S3: Chlorination of CLO at different pHs. Experimental conditions: [CLO]_0_ = 0.1 mg L^−1^; Figure S4: Effect of carbonate (a), nitrate (b), dissolved organic matter (c) and real water (d) on the chlorination of CLO; Figure S5: NO_3_^−^ formation at different pHs. Experimental conditions: [Cl_2_]_0_ = 10 mg L^−1^, [CLO]_0_ = 0.1 mg L^−1^; Figure S6: Species distribution of haloacetic acids (HAAs) at different pHs. Experimental conditions: [Cl_2_]_0_ = 10 mg L^−1^, [CLO]_0_ = 0.1 mg L^−1^; Figure S7: CP1 fragmentation analysis (a), MS spectrum (b) and MS^2^ spectrum (c); Figure S8: CP2 fragmentation analysis (a), MS spectrum (b) and MS^2^ spectrum (c); Figure S9: CP3 fragmentation analysis (a), MS spectrum (b) and MS^2^ spectrum (c).
