# Supplementary material for: Chlorination of Clothianidin During Disinfection: Kinetics, Pathways, and Toxicity
Source: Toxics. 2026 May 22;14(6):453. doi: 10.3390/toxics14060453 (PMC13306777; doi:10.3390/toxics14060453)
Supplement: Supplementary file 1 [file toxics-14-00453-s001.zip › toxics-4266080-supplementary.pdf]

Supplementary Information

Chlorination of clothianidin during disinfection: kinetics, pathways, and toxicity

Fang Wei [a,#], Lei Wu [b,#], Fei Meng [c], Sanyan Du [d], Xinyuan Wu [e], Jun Hu [e,*]

[a] *School of Hydraulic Engineering, Zhejiang Tongji Vocational College of Science and Technology, Hangzhou, China*

[b] *Longyou Inspection & Testing Research Institute, Quzhou, China*

[c] *Zhejiang Yilong Environmental Protection Technology Co., Ltd., Hangzhou, Zhejiang, China*

[d] *Yangtze River Delta Institute of Health Agriculture (Zhejiang) Co., Ltd., Jiaxing, Zhejiang, China*

[e] *College of Environment, Zhejiang University of Technology, Hangzhou, China*

# Co-first authors.

These authors contributed equally to this work.

[*] *Corresponding author.*

e-mail address: hujun1988@zjut.edu.cn (J. Hu)

Text S1 Chronic cytotoxicity assay

Compared to other experiments, the dosages of neonicotinoid pesticides (NPs) and free chlorine (Cl_2) in this section were increased by 1000 times for convenience in quantifying reaction products. After 36 h of reaction, the solution was scavenged by an excess of sodium thiosulfate and freeze-dried in a vacuum. The residues were extracted with methanol in a Soxhlet extractor. Finally, methanol was evaporated with a gentle stream of nitrogen and the extracted products were quickly quantified. For comparison, a control sample was prepared in the same way without the addition of Cl_2.

Chinese Hamster Ovary (CHO) cells were cultivated in Gibco MEM medium (5.0 mL) containing 10% foetal bovine serum (FBS) in a culture flask (25 cm^3) under humid incubation conditions (37 °C, 5% CO_2). The medium was renewed every two days, and the cells were split by using 0.25% trypsin-ethylenediamine tetraacetic acid (1.0 mL) (BioPike, China) solution.

The cultured cells were seeded in a 96-well plate with at the density of 2.0×10^3 cell/well for a total volume of 100 μL. After 48 h, the cells were treated by a series of diluted concentrations (by 200–50000 times) of filtered waters or dimethylsulfoxide (DMSO) (0.1%) prepared in Gibco MEM medium containing 1% FBS. After 72 h, the reaction was spiked with 10 μL of CCK-8 solution (Dojindo, Japan) and continued for 2 h. The absorbance for cell viability determination was read on a microplate reader (Potenov PT-3502, China) at 450 nm. The cell proliferation ratio (CPR) was calculated by obtained cell viability to evaluate the effect of chlorination on the chronic cytotoxicity of NPs.

$$\mathrm{CPR} = \left(\frac{\mathrm{OD}_{\mathrm{exp}}}{\mathrm{OD}_{\mathrm{con}}}\right) \times 100\%$$

where OD_{exp} is the absorbance of experimental groups (treated by diluted filtered waters), and OD_{con} is the absorbance of control groups (treated by 0.1% DMSO), respectively.

Text S2 Instrumental conditions for chemical analysis

Clothianidin (CLO). CLO was analyzed by a Waters Xevo TQ-s ultra-high-performance liquid chromatograph integrated with a tandem mass spectrometer (UPLC) and a YMC-Pack ODS-AQ column (2.1 mm × 100 mm, 3.0 μm). The mobile phase included acetonitrile (phase A) and 0.01% formic acid (phase B). The flow rate of mobile phase was 0.3 mL min^{-1}. The A/B ratio (v/v) held for 1 min at 100%/0%, decreased linearly to 90%/10% within 1 min, decreased linearly to 10%/90% within 1 min and held for 1 min, and finally increased linearly to 100%/0% within 1 min and held for 1 min. The electrospray ionization (ESI) ion source was used to conduct multiple reaction monitoring (MRM) in the positive ion mode, and two transition ions were used to conduct quantitative and qualitative analysis of samples. Nitrogen and argon were employed as the carrier gas and collision gas, respectively. The gas flow rate was 1.0 mL min^{-1}. The ionization source temperature was 300 °C. The capillary and cone voltages were 2600 and 48 V, respectively.

Nitrate (NO_3^-), nitrite (NO_2^-) and ammonium (NH_4^+). NO_3^- and NO_2^- were analyzed by a Dionex Aquion RFIC ion chromatograph (USA) with an IonPac AS19 column (250 mm × 4.0 mm, 5.0 μm). KOH solution (20 mM) was used as eluent at a flow rate of 1.0 mL min^{-1}. NH_4^+ was quantified by an ion chromatograph (Dionex ICS-3000) with an IonPac CS12A column (250 mm × 4.0 mm, 5.0 μm). The eluent was methanesulfonic acid (20 mM) at a flow rate of 1.0 mL min^{-1}.

Trihalomethanes (THMs) and haloacetic acids (HAAs). THMs and HAAs were analyzed by a gas chromatograph integrated with an electron capture detector (GC/ECD, Agilent 6890 N, USA) and a DB-5 ms capillary column (30 m × 0.25 mm, 0.25 μm). The instrumental conditions for THM analysis were as follows: injection volume of 1.0 μL (splitless mode), helium gas flow rate of 2.0 mL min^{-1}, injector temperature of 210 °C, detector temperature of 300 °C, and initial oven temperature held at 40 °C for 5 min, ramped to 240 °C at 10 °C min^{-1} and held for 2 min. For HAAs, the instrumental conditions were as follows: injector temperature of 210 °C, detector temperature of 300 °C, and initial oven temperature held at 40 °C for 4 min, and ramped to 80 °C at 2 °C min^{-1}.

Chlorination products (CPs). The CPs were first concentrated by Oasis HLB assay cartridges (200 mg, 6 mL, 30 μm). Before sample loading, each reagent cartridge was adjusted with 5 mL of methanol, and then washed once with 5 mL of Milli-Q water. Afterwards, each sample was extracted through a filter cartridge at a flow rate of 1.0 mL min^{-1} under vacuum. After loading the sample, the filter cartridge was washed with 5 mL of Milli-Q water, and then the intermediate products were eluted with 1.5 mL of acetonitrile/water (90%/10%, v/v) and 0.1% formic acid. The eluent was filtered with a glass fiber membrane (0.22 μm), then transferred to an injection vial and finally stored at −20 °C.

The products were analyzed by an ultra-performance liquid chromatograph coupled with a quadrupole time-of-flight mass spectrometer (UPLC-QTOF-MS/MS, Waters ACQUITY UPLC I-Class, Waters SYNAPT XS HDMS, USA) and a ACQUITY BEH C18 chromatographic column (2.1 mm × 1000 mm, 1.7 μm). Ammonium formate in water (5 mM) and methanol were used as the mobile phases A and B, respectively. The A/B ratio (v/v) was maintained at 95%/5% for the initial 2 min, linearly increased to 55%/45% over 9 min, further adjusted to 25%/75% over 1 min, then raised to 5%/95% over 1 min, and finally held at 5%/95% for 2 min. The flow rate was 0.3 mL min^{-1}. The MS data were acquired on a Waters SYNAPT XS HDMS system by Masslynx V4.2 software (Waters), using ESI positive ion mode and two acquisition methods: the first is MS^E mode. MS^E is a novel, patented mode of data acquisition that permits the seamless collection of a comprehensive catalogue of information for both precursor and fragment ions in a single analysis. This is achieved by rapidly alternating between two functions: an accurate mass measurement of precursor ions obtained at low collision energy. The other one is to provide precise fragment ion mass at high energy. MSE settings include low collision energy (6 eV) for MS and ramped high energy (15–40 eV) for fragment ion generation. The second is MSMS mode. It can collect fragment ions of target ions, and different compounds at 15–40 V collision energy to collect data. The QTOF mass spectrometer is calibrated for mass axis and lock mass setup before data acquisition, and the lock mass is continuously calibrated in real time during the actual acquisition process. The mass calibration range was m/z 50–1200 (calibration solution was sodium formate and leucine enkephalin was

used as the lock mass reference for real-time calibration).

Unknown compound identification was accomplished via a non-targeted screening process in the UNIFI software (Waters) in the waters_connect platform. The process was based on a raw peak detection algorithm and compared to a control sample. The screening threshold was set at 5-fold higher candidate ion response values than the control sample to exclude low-abundance degradation products. Candidate ions that passed the threshold screening generated extracted ion chromatograms (EIC, mass extraction window 10 mDa) and were filtered for compounds with intensities >100 counts. The software automatically matches the primary and secondary spectra of the compounds based on local and online databases, and the SmartScore function of the UNIFI software calculates fragment ion matches using customized parameters, with the score set at 16, the hydrogen atom difference at 3, the fragment mass bias set at 3 mDa, and the maximum number of bond breaks permitted at four. The precise masses of precursor ions (mass error < 3 mDa) and daughter ions (mass error < 3 mDa) are finally evaluated and compared to the experimental data with theoretical isotopic ratio differences. Final validation was accomplished by manually checking the molecular formula and fragmentation spectra against the cleavage pattern of the parent of CLO and THI.

Reference

Noestheden, M., Roberts, S., Hao, C.Y., 2016. Nitenpyram degradation in finished drinking water. Rapid Commun. Mass Spectrom. 30, 1653–1661.

Feng, X.X., Xu, W. Z., Ji, X.M., Liang, J.F., Liu, X.Y., Liu, X.K., Liu, C.G., Qu, G.B., Liu, R.Z., 2024. First evidence of novel organothiophosphate esters as prevalent new pollutants in dust from automotive repair shops discovered by high-resolution mass spectrometry. Environ. Sci. Technol. 58, 22790−22798.

Text S3 Determination of reaction order (*m*)

The pseudo-second-order kinetics of CLO chlorination is explained as follows:

$$-\frac{d[CLO]}{dt} = k_{obs}[CLO]_T = k_{app}[Cl_2]_T[CLO]_T$$

$$k_{obs} = k_{app}[Cl_2]_T^m$$

$$\ln(k_{obs}) = \ln(k_{app}) + m\ln([Cl_2]_T)$$

where m is the slope of $\ln k_{obs}$ values to $\ln[Cl]_T$ values.

Text S4 pH-dependent reaction kinetics of CLO chlorination

The second-order reaction of CLO chlorination can be expressed as below:

$$[\mathrm{CLO}^+] \approx [\mathrm{CLO}]_\mathrm{T}$$

$$[\mathrm{HOCl}] + [\mathrm{OCl}^-] \approx [\mathrm{Cl}_2]_\mathrm{T}$$

$$\begin{aligned} -\frac{\mathrm{d}[\mathrm{CLO}]}{\mathrm{dt}} &= k_{\mathrm{HOCl,CLO}^+}[\mathrm{CLO}^+][\mathrm{HOCl}] + k_{\mathrm{OCl}^-,\mathrm{CLO}^+}[\mathrm{CLO}^+][\mathrm{OCl}^-] \\ &= k_{\mathrm{HOCl,CLO}^+}[\mathrm{CLO}]_\mathrm{T}[\mathrm{HOCl}] + k_{\mathrm{OCl}^-,\mathrm{CLO}^+}[\mathrm{CLO}]_\mathrm{T}[\mathrm{OCl}^-] \\ &= (k_{\mathrm{HOCl,CLO}^+}[\mathrm{HOCl}] + k_{\mathrm{OCl}^-,\mathrm{CLO}^+}[\mathrm{OCl}^-])[\mathrm{CLO}]_\mathrm{T} \end{aligned}$$

To find a different expression for speciation coefficient, the acid constant of HOCl was defined as follows:

$$K_\mathrm{a}^\mathrm{HOCl} = \frac{[\mathrm{OCl}^-][\mathrm{H}^+]}{[\mathrm{HOCl}]} \Rightarrow [\mathrm{OCl}^-] = \frac{K_\mathrm{a}^\mathrm{HOCl}[\mathrm{Cl}_2]_\mathrm{T}}{K_\mathrm{a}^\mathrm{HOCl} + [\mathrm{H}^+]} \Rightarrow [\mathrm{HOCl}] = \frac{[\mathrm{H}^+][\mathrm{Cl}_2]_\mathrm{T}}{K_\mathrm{a}^\mathrm{HOCl} + [\mathrm{H}^+]}$$

Given this, the expression of the second-order reaction can be converted to the below:

$$-\frac{\mathrm{d}[\mathrm{CLO}]}{\mathrm{dt}} = \frac{k_{\mathrm{HOCl,CLO}^+}[\mathrm{H}^+] + k_{\mathrm{OCl}^-,\mathrm{CLO}^+}K_\mathrm{a}^\mathrm{HOCl}}{K_\mathrm{a}^\mathrm{HOCl} + [\mathrm{H}^+]}[\mathrm{CLO}]_\mathrm{T}[\mathrm{Cl}_2]_\mathrm{T}$$

The k_{app} can be finally formulated as follows:

$$k_\mathrm{app} = \frac{k_{\mathrm{HOCl,CLO}^+}[\mathrm{H}^+] + k_{\mathrm{OCl}^-,\mathrm{CLO}^+}K_\mathrm{a}^\mathrm{HOCl}}{K_\mathrm{a}^\mathrm{HOCl} + [\mathrm{H}^+]}$$

Table S1 Major characteristics of real waters post 0.22 μm membrane filtration.

Real Water	pH [a]	DOC (mg L^{-1}) [a]	$SUVA_{254}$ (L mg^{-1} m^{-1}) [b]
Huzhou (HZ)	8.23 (0.04)	2.597 (0.018)	0.47 (0.01)
Nanchang (NC)	8.31 (0.05)	5.924 (0.042)	0.49 (0.02)
Jinhua (JH)	8.18 (0.05)	6.829 (0.021)	0.45 (0.01)

[a] Mean (standard deviation), n = 3; [b] $SUVA_{254} = UV_{254}/DOC$.

Table S2 Identified products of clothianidin (CLO).

Products	m/z	Formula	Structure
CP1	206.0142	$C_6H_8ClN_3OS$	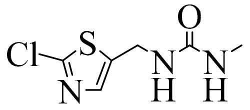
CP2	148.9927	$C_4H_5ClN_2S$	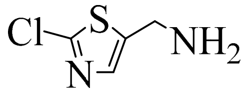
CP3	236.9838	$C_5H_5ClN_4O_3S$	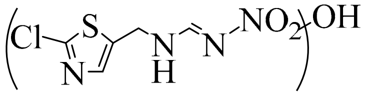

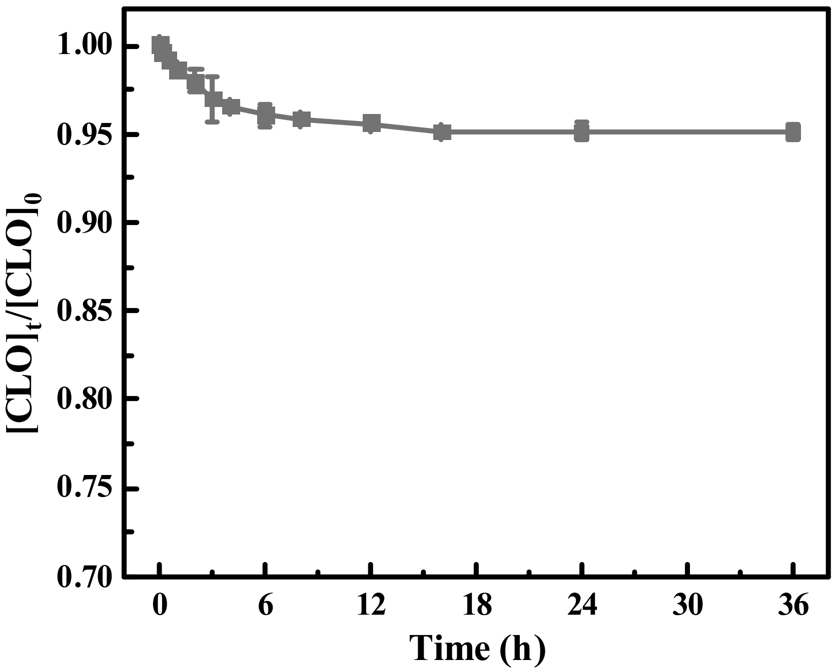

Fig. S1. Hydrolysis of clothianidin (CLO). Experimental conditions: $[NPs]_0$ = 0.1 mg L^{-1}, pH = 7.0.

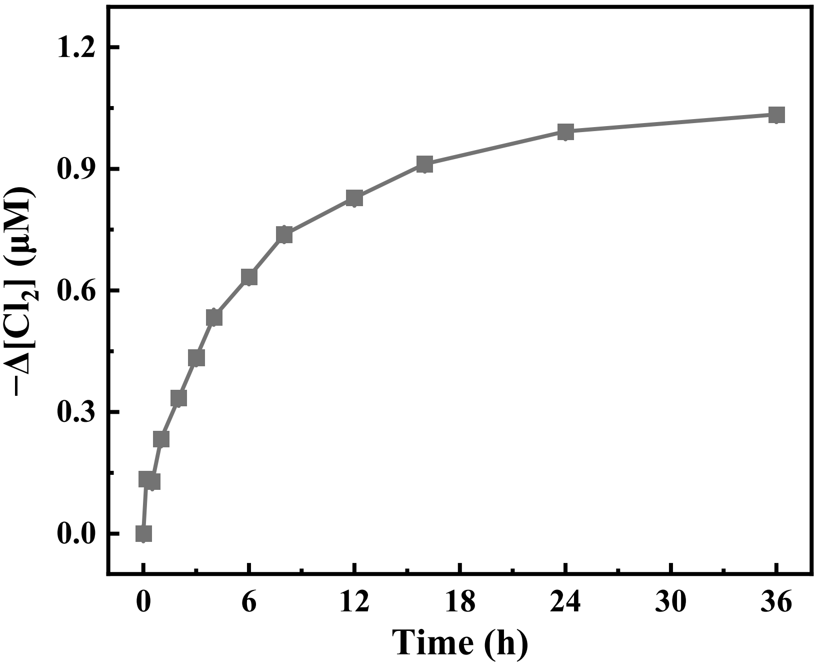

Fig. S2. Chlorine consumption during CLO chlorination. Experimental conditions: $[Cl_2]_0$ = 10 mg L^{-1} (0.14 mM), $[CLO]_0$ = 0.1 mg L^{-1}, pH = 7.0.

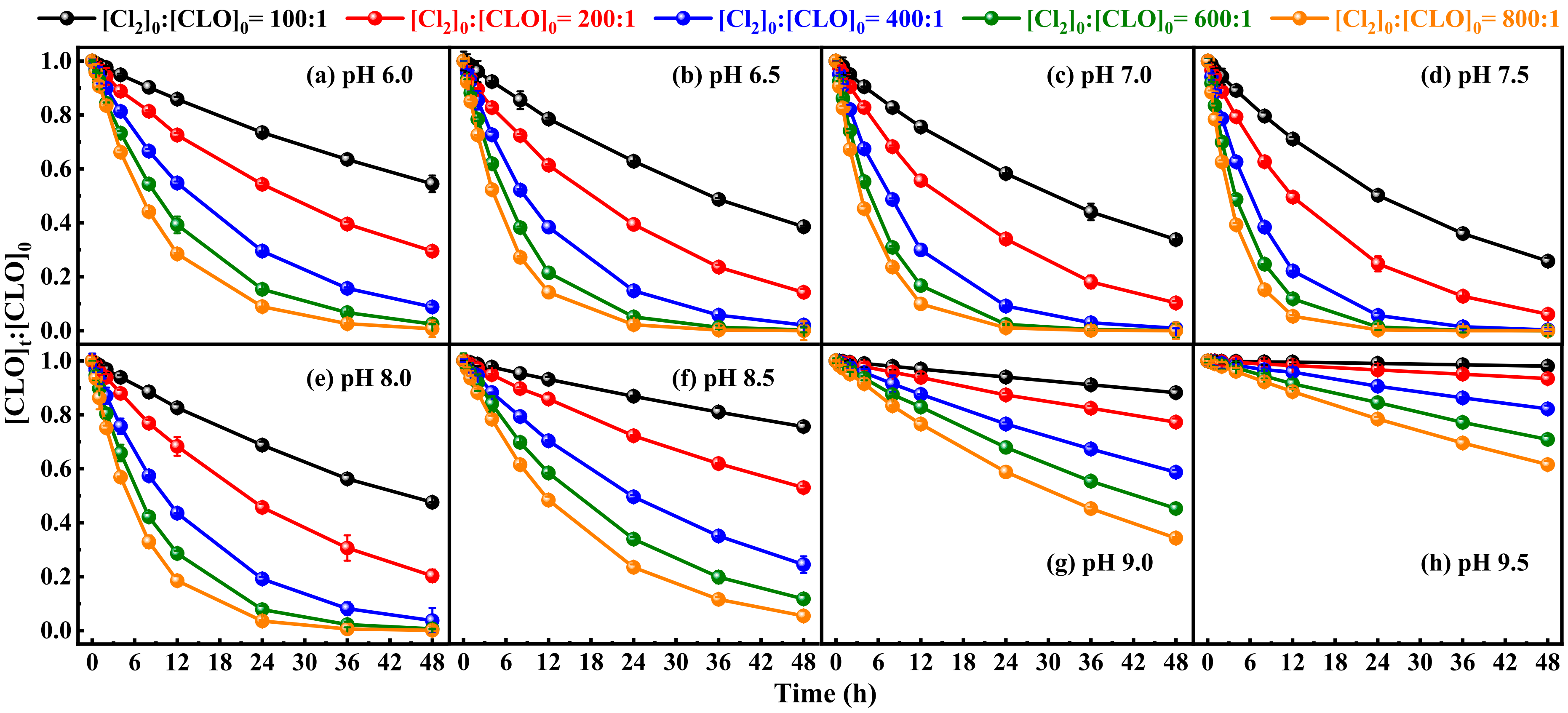

Fig. S3. Chlorination of CLO at different pHs. Experimental conditions: $[CLO]_0 = 0.1$ mg L^{-1}.

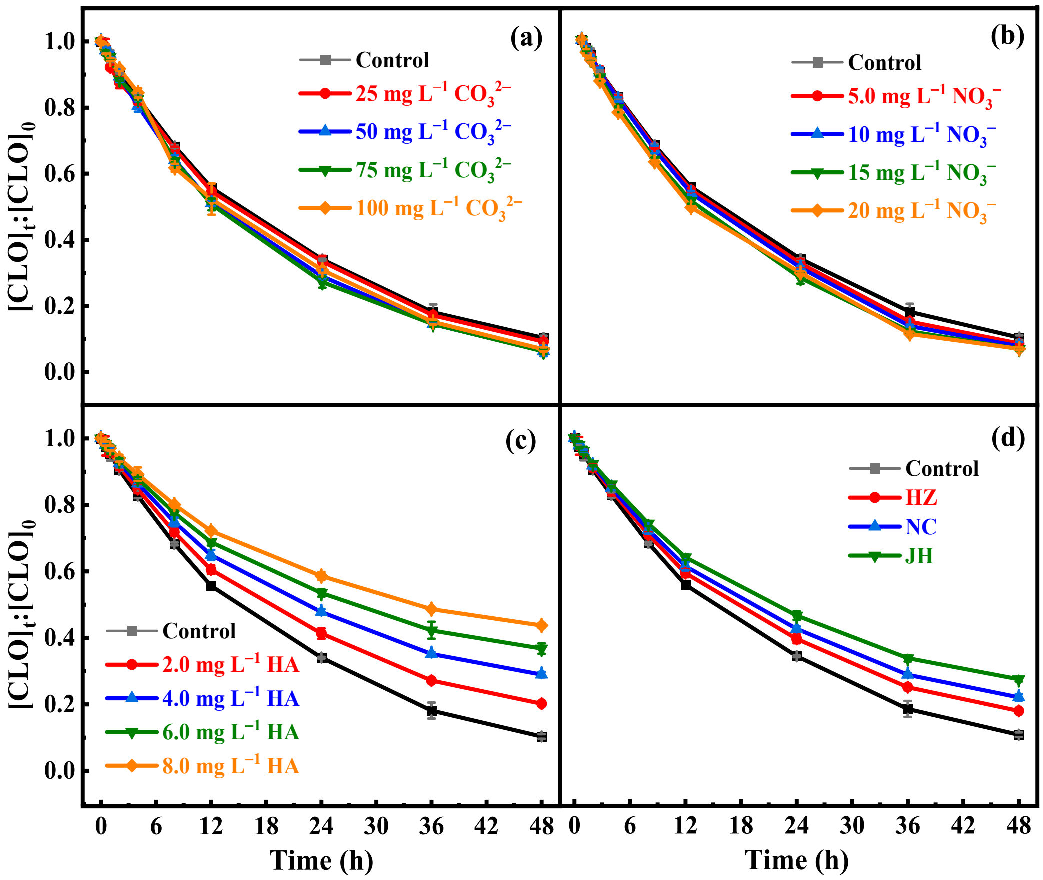

Fig. S4. Effect of carbonate (a), nitrate (b), dissolved organic matter (c) and real water (d) on the chlorination of CLO.

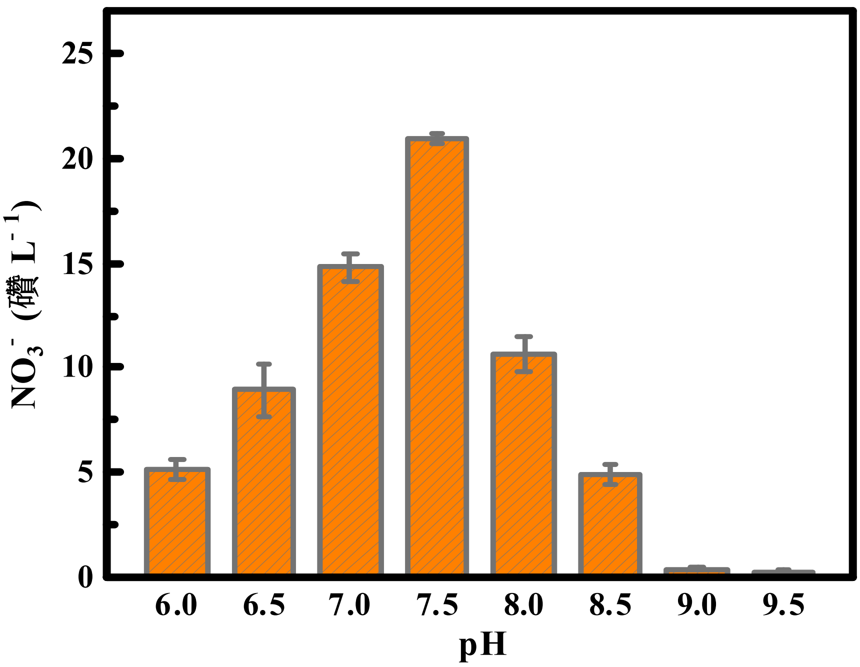

Fig. S5. NO_3^- formation at different pHs. Experimental conditions: $[Cl_2]_0$ = 10 mg L^{-1}, $[CLO]_0$ = 0.1 mg L^{-1}.

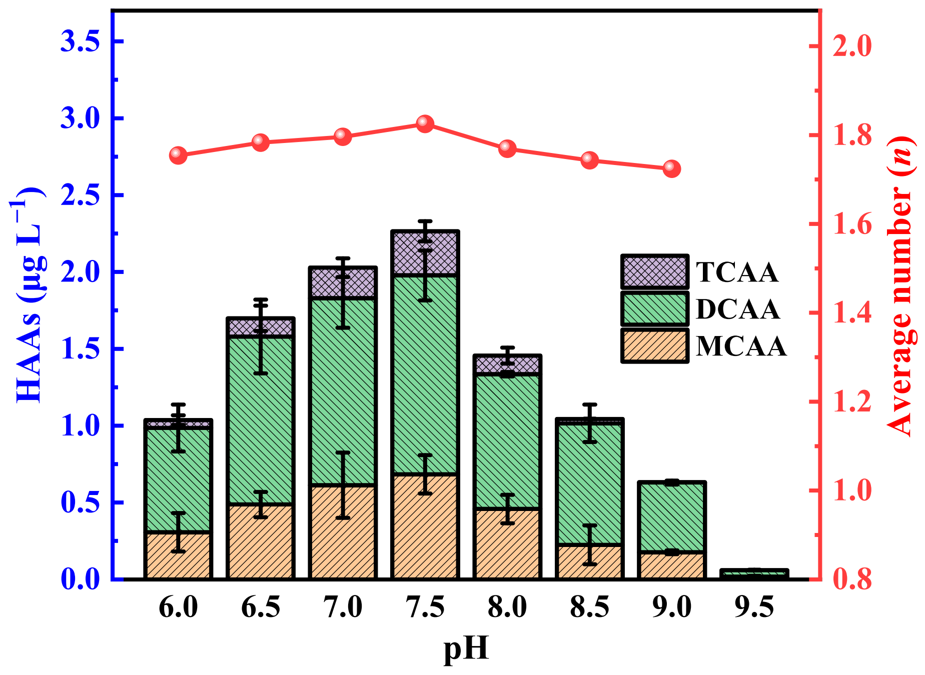

Fig. S6. Species distribution of haloacetic acids (HAAs) at different pHs. Experimental conditions: $[Cl_2]_0 = 10$ mg L^{-1}, $[CLO]_0 = 0.1$ mg L^{-1}.

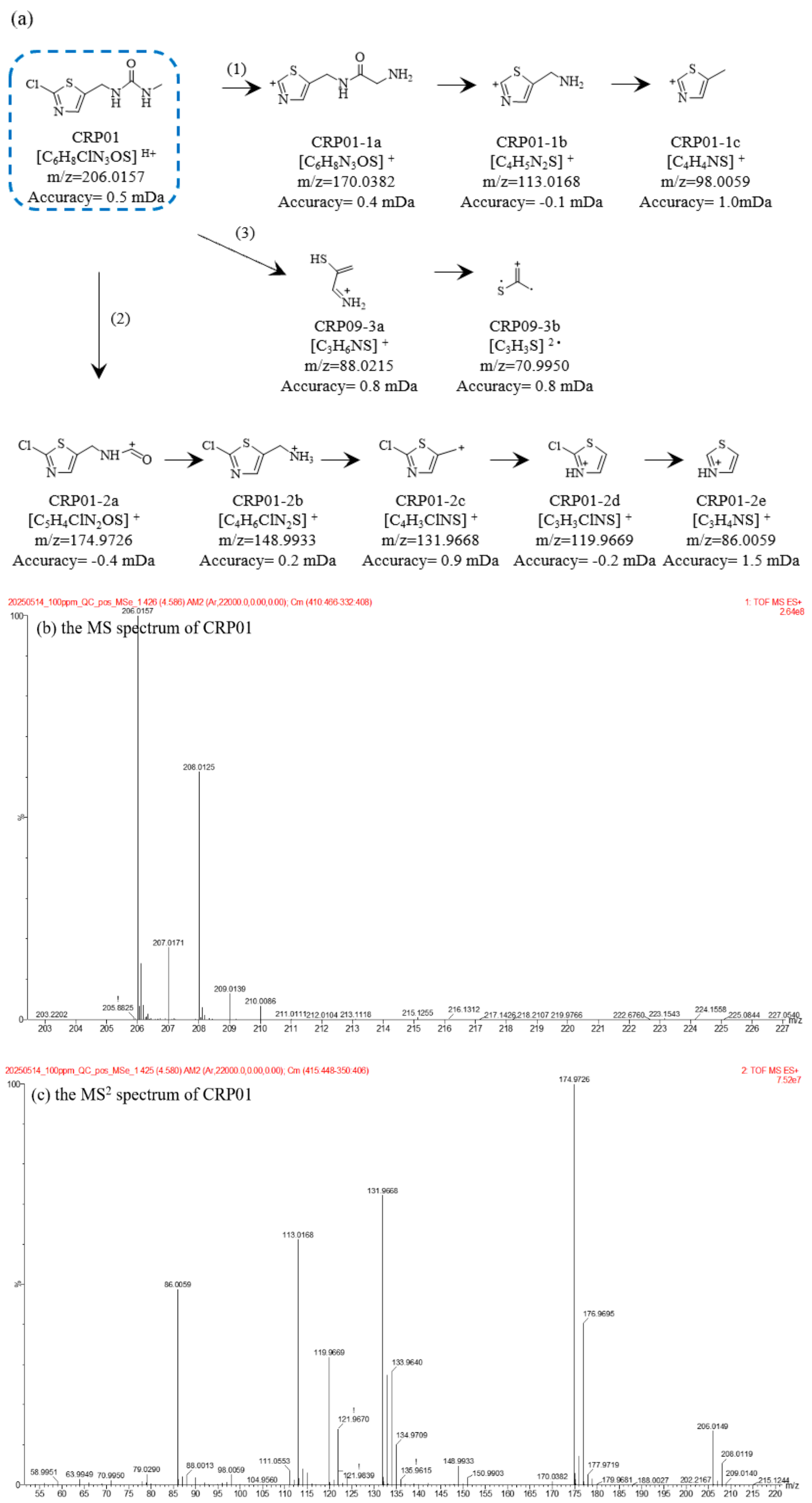

Fig. S7. CP1 fragmentation analysis (a), MS spectrum (b) and MS^2 spectrum (c). Fragmentation pathway analysis: Firstly, the chlorine atom is eliminated, yielding the

fragment with m/z 170.0382. Subsequently, the fragment with m/z 170.0382 eliminates the carbonyl-aminomethyl group to form the fragment with m/z 113.0168, and then further eliminates an amino group to generate the fragment with m/z 98.0059. Secondly, an aminomethyl group is eliminated, resulting in the fragment with m/z 174.9726. Following this, cleavage of the carbonyl group occurs, producing the fragment with m/z 148.9933. This fragment then eliminates an amino group to form the fragment with m/z 131.9668, which further eliminates a methyl group to yield the fragment with m/z 119.9669. Finally, the fragment with m/z 119.9669 eliminates a chlorine atom, generating the fragment with m/z 86.0059. Thirdly, the parent molecule eliminates the carbonyl-aminomethyl group; a ring-opening reaction occurs in the chlorinated thiazole ring moiety, with the ring opening taking place at the C-S and C-N bonds, resulting in the fragment with m/z 88.0215. This fragment then eliminates an amino group to form the fragment with m/z 70.9950.

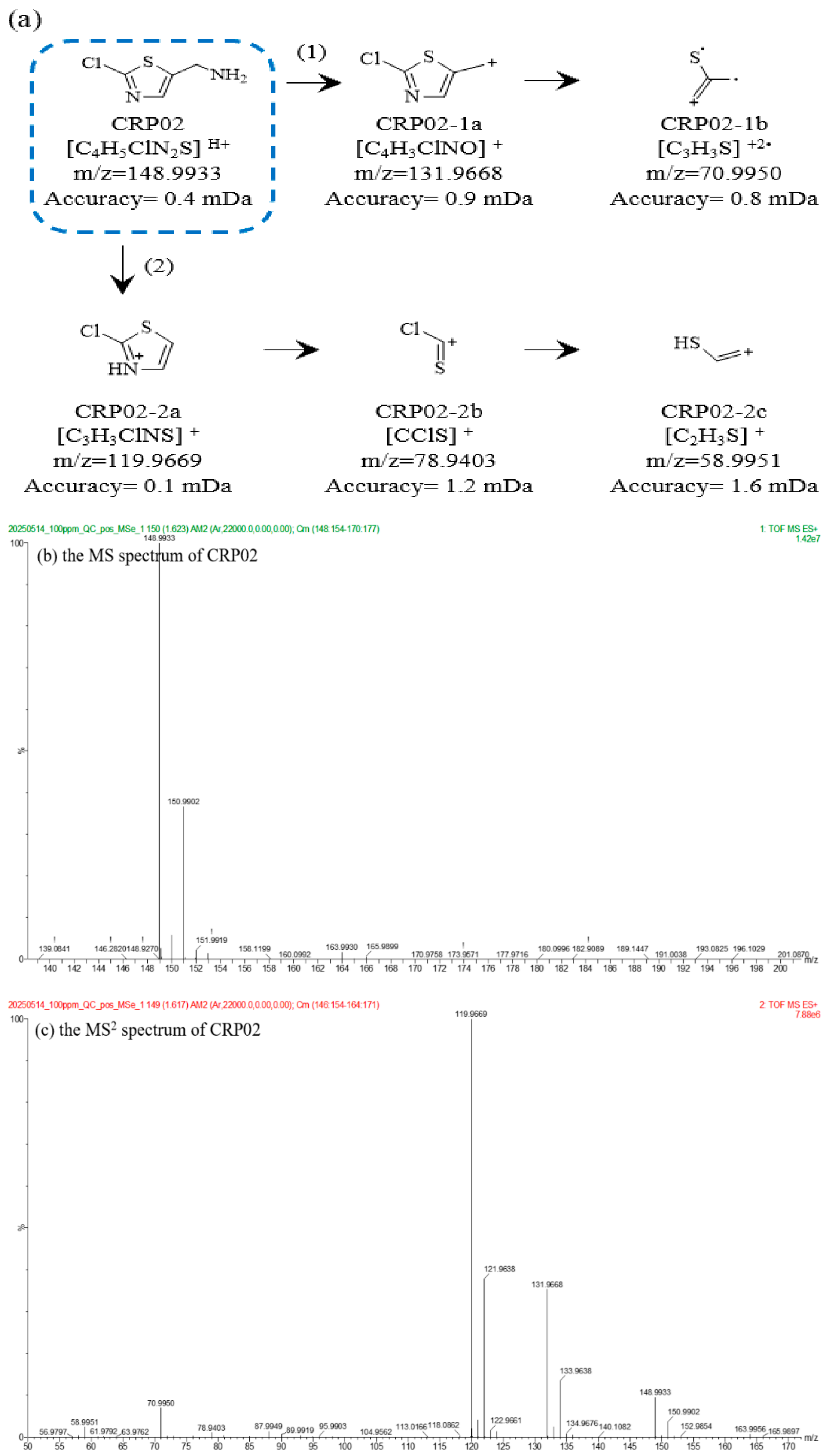

Fig. S8. CP2 fragmentation analysis (a), MS spectrum (b) and MS^2 spectrum (c). Fragmentation pathway analysis: Firstly, the amino group is eliminated, yielding the fragment with m/z 131.9668. Subsequently, the thiazole ring undergoes a ring-opening

reaction, with the ring opening occurring between the S-C bond and C-N bond, resulting in the fragment with m/z 70.9950. Secondly, the parent molecule eliminates the aminomethyl group to yield the fragment with m/z 119.9669. The thiazole ring then undergoes a ring-opening reaction, with the ring opening located at the S-C bond and C=N bond, producing the fragment with m/z 78.9403. It then further eliminates a chlorine atom to yield the fragment with m/z 58.9951.

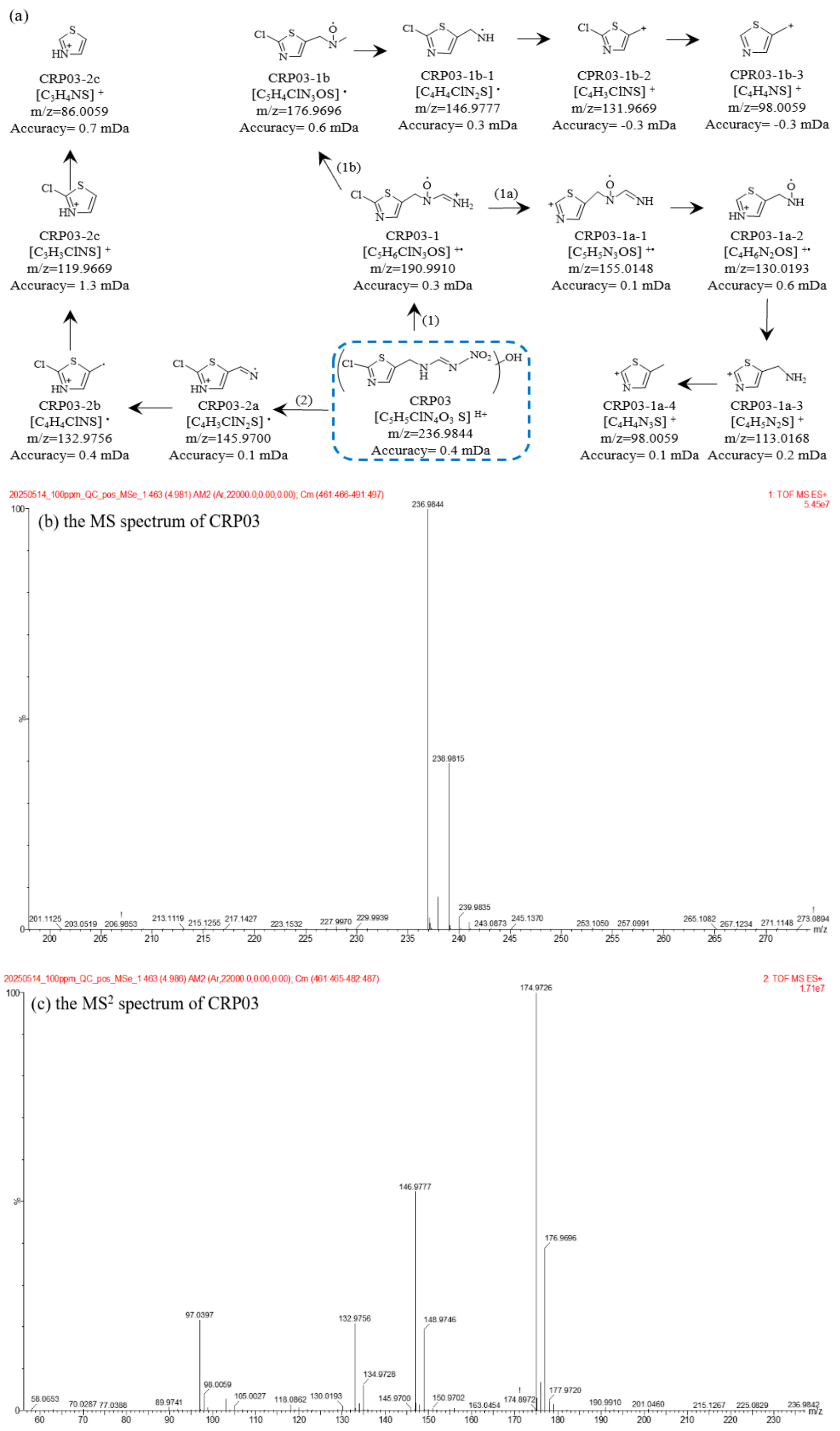

Fig. S9. CP3 fragmentation analysis (a), MS spectrum (b) and MS^2 spectrum (c). Fragmentation pathways analysis: Firstly, the nitro group is eliminated, yielding the fragment with m/z 190.9910. The fragment with m/z 190.9910 undergoes

dehydrogenation and dechlorination to yield the fragment with m/z 155.0148. Subsequently, it eliminates the -CHNH group to form the fragment with m/z 130.0193, followed by deoxygenation to generate the fragment with m/z 113.0168, and finally eliminates the amino group to yield the fragment with m/z 98.0059. The fragment with m/z 190.9910 eliminates the amino group to yield the fragment with m/z 176.9889. It then eliminates an oxygen atom and a methyl group to form the fragment with m/z 146.9777, followed by elimination of the amino group to generate the fragment with m/z 131.9669, and finally eliminates the chlorine atom to yield the fragment with m/z 98.0059. Secondly, the hydroxyl group and the -CH=N-NO_2-NH moiety within the 2-nitroguanidine structure are eliminated, yielding the fragment with m/z 145.9700. This fragment then undergoes homolytic cleavage to eliminate the nitrogen radical, resulting in the intermediate with m/z 132.9756. Subsequently, it undergoes a methyl elimination reaction to generate the fragment with m/z 119.9669, and finally undergoes a dechlorination reaction to yield the final product fragment with m/z 86.0059.
